# *FBXO32*, encoding a member of the SCF complex, is mutated in dilated cardiomyopathy

**DOI:** 10.1186/s13059-015-0861-4

**Published:** 2016-01-11

**Authors:** Nadya Al-Yacoub, Ranad Shaheen, Salma Mahmoud Awad, Muhammad Kunhi, Nduna Dzimiri, Henry C. Nguyen, Yong Xiong, Jehad Al-Buraiki, Waleed Al-Habeeb, Fowzan S. Alkuraya, Coralie Poizat

**Affiliations:** Cardiovascular Research Program, King Faisal Specialist Hospital & Research Centre, Riyadh, 11211 Saudi Arabia; Department of Genetics, King Faisal Specialist Hospital & Research Centre, Riyadh, 11211 Saudi Arabia; Cardiovascular & Pharmacogenetics, Genetics Department, King Faisal Specialist Hospital & Research Centre, Riyadh, 11211 Saudi Arabia; Department of Molecular Biophysics and Biochemistry, Yale University, New Haven, CT 06520 USA; Heart Centre, King Faisal Specialist Hospital & Research Centre, Riyadh, 11211 Saudi Arabia; King Saud University, Riyadh, 11211 Saudi Arabia

**Keywords:** Familial dilated cardiomyopathy, novel mutation, FBXO32

## Abstract

**Background:**

Dilated cardiomyopathy (DCM) is a common form of cardiomyopathy causing systolic dysfunction and heart failure. Rare variants in more than 30 genes, mostly encoding sarcomeric proteins and proteins of the cytoskeleton, have been implicated in familial DCM to date. Yet, the majority of variants causing DCM remain to be identified. The goal of the study is to identify novel mutations causing familial dilated cardiomyopathy.

**Results:**

We identify *FBXO32 (ATROGIN 1)*, a member of the F-Box protein family, as a novel DCM-causing locus. The missense mutation affects a highly conserved amino acid and is predicted to severely impair binding to SCF proteins. This is validated by co-immunoprecipitation experiments from cells expressing the mutant protein and from human heart tissue from two of the affected patients. We also demonstrate that the hearts of the patients with the FBXO32 mutation show accumulation of selected proteins regulating autophagy.

**Conclusion:**

Our results indicate that abnormal SCF activity with subsequent impairment of the autophagic flux due to a novel *FBXO32* mutation is implicated in the pathogenesis of DCM.

**Electronic supplementary material:**

The online version of this article (doi:10.1186/s13059-015-0861-4) contains supplementary material, which is available to authorized users.

## Background

Dilated cardiomyopathy (DCM) is a primary myocardial disease characterized by dilatation and impaired systolic function of one or both ventricles. DCM is often associated with atrial and /or ventricular arrhythmias and sudden death which can occur at any stage of the disease [1]. DCM is a common cause of progressive heart failure and the most frequent indication for cardiac transplantation [2, 3]. The prevalence of DCM has been estimated to be 1 in 2,500 individuals for idiopathic DCM although recent sequencing data suggest that this number is largely underestimated, as many patients are pre-symptomatic [4]. Among patients with idiopathic DCM 20–50 % of the cases are familial (FDC) [5]. FDC is genetically heterogeneous, with mutation in more than 30 genes reported to date [6].

The overlap between the genes mutated in hypertrophic cardiomyopathy (HCM) and DCM makes it challenging to establish clear genotype/phenotype correlation in DCM [6, 7]. The vast majority of DCM-causing mutations are rare or even private [8, 9]. However, the mutations reported so far within the known genes are only responsible for one-third of the genetic causes of DCM; so the causal mutations in the majority of DCM patients remain unknown [4].

The ubiquitin-proteasome system (UPS) is responsible for the targeted degradation of proteins regulating pivotal signaling pathways in the heart [10]. Dysregulation of the UPS can lead to major pathologies. For instance, hyper-ubiquitination of proteins and increased expression of E1 (ubiquitin-activating) and E2 (ubiquitin-conjugating) enzymes have been reported in explants from DCM patients [11]. The E3 ligase is a key enzyme in the UPS as it recognizes specific protein substrates that undergo ubiquitin conjugation [12].

FBXO32(MAFbx1/ Atrogin1) is a cardiac and muscle specific F-Box protein with E3 ligase activity that localizes at the sarcomere. FBXO32 is a component of the SCF complex, which also includes SKP1, CUL1, and ROC1. The protein serves as an adaptor that targets specific substrates for canonical- or non-canonical ubiquitination. FBXO32 was initially described as a key mediator of skeletal muscle atrophy [13, 14]. Because of its role in muscle atrophy, FBXO32 was expected to act as a negative regulator of hypertrophy in heart muscle. However, gain and loss of function studies in mice have produced mixed phenotypes leaving open questions on the functional role of FBXO32 in cardiac muscle [13, 15]. FBXO32 negatively regulates physiological cardiac hypertrophy by enhancing the activity of Forkhead transcription factors [16]. Under stress conditions, FBXO32 is also a negative regulator of pathological cardiac hypertrophy, which is associated with degradation of calcineurin A [13]. However, FBXO32 was also shown to be a mediator of pathological cardiac remodeling via stabilization of IκB-α [15]. In a rat model of chronic heart failure after myocardial infarction, TNF-alpha induces troponin I degradation through an FBXO32/Murf1-dependent pathway [16]. Most recently, a role of FBXO32 in myocardial aging was reported as FBXO32 deficiency led to cardiomyopathy over time due to impaired autophagy [17].

In the present study, we describe a family with FDC that did not link to previously known DCM genes, and identify *FBXO32* as a new member of genes causing FDC. We describe the first homozygous missense mutation in FBXO32 that abrogates interaction with members of the SCF complex, and is associated with defects in proteins regulating the autophagy/lysosome machinery.

## Results

### Identification of a family with DCM

The family was a consanguineous Saudi family consisting of healthy parents who were first cousins, four children with DCM, and six healthy siblings. FDC was suspected based on the family history of DCM, which was confirmed after cardiovascular screening of at-risk family members. There was also a family history of heart transplantation as three of the affected siblings (participants IV.5, IV.7, and IV.8) underwent cardiac transplantation. Patient IV.4 was the first to be diagnosed with DCM and was a candidate for heart transplantation, which he rejected when his condition deteriorated. The patient had a severe dilatation of all the cardiac chambers. Systolic function was severely impaired and the ventricle was hypertrabeculated. There was evidence of severe valve regurgitation and elevated right ventricular (RV) systolic pressure. Details of the clinical features of the family are provided in Table S1 in Additional file 1.

The index (Patient IV.5) was diagnosed with DCM during family screening. Echocardiography revealed a moderately-to-severely dilated left ventricle (LV) with hypertrabeculated cavities, and severely dilated left atria (LA) (Figure S1 in Additional file 2), elevated filling pressures and low cardiac output. LV function was severely impaired with a pronounced global hypokinesis. The RV was mildly dilated with severely reduced systolic function. Like patient IV.4, the LA was severely dilated. Three years post transplant the patient showed no sign of recurrence of the disease.

Patient IV.7 was diagnosed with DCM during family screening. The patient had multiple recurrent admissions with decompensated heart failure. Echocardiography showed a severe dilatation of the LV and LA associated with a severely reduced ejection fraction (Figure S1 in Additional file 2). LV filling pressure raised and there was evidence of moderate tricuspid regurgitation after which the patient was transplanted.

Patient IV.8 was also diagnosed with DCM and underwent cardiac transplantation after a deterioration of his condition. Echocardiography prior to the cardiac transplantation showed a severe dilatation of the LV and a severely reduced LV systolic pressure and global hypokinesis. LA was severely dilated while RA was moderately to severely dilated.

### Homozygosity mapping and linkage analysis identify a novel DCM locus

**Fig. 1:**
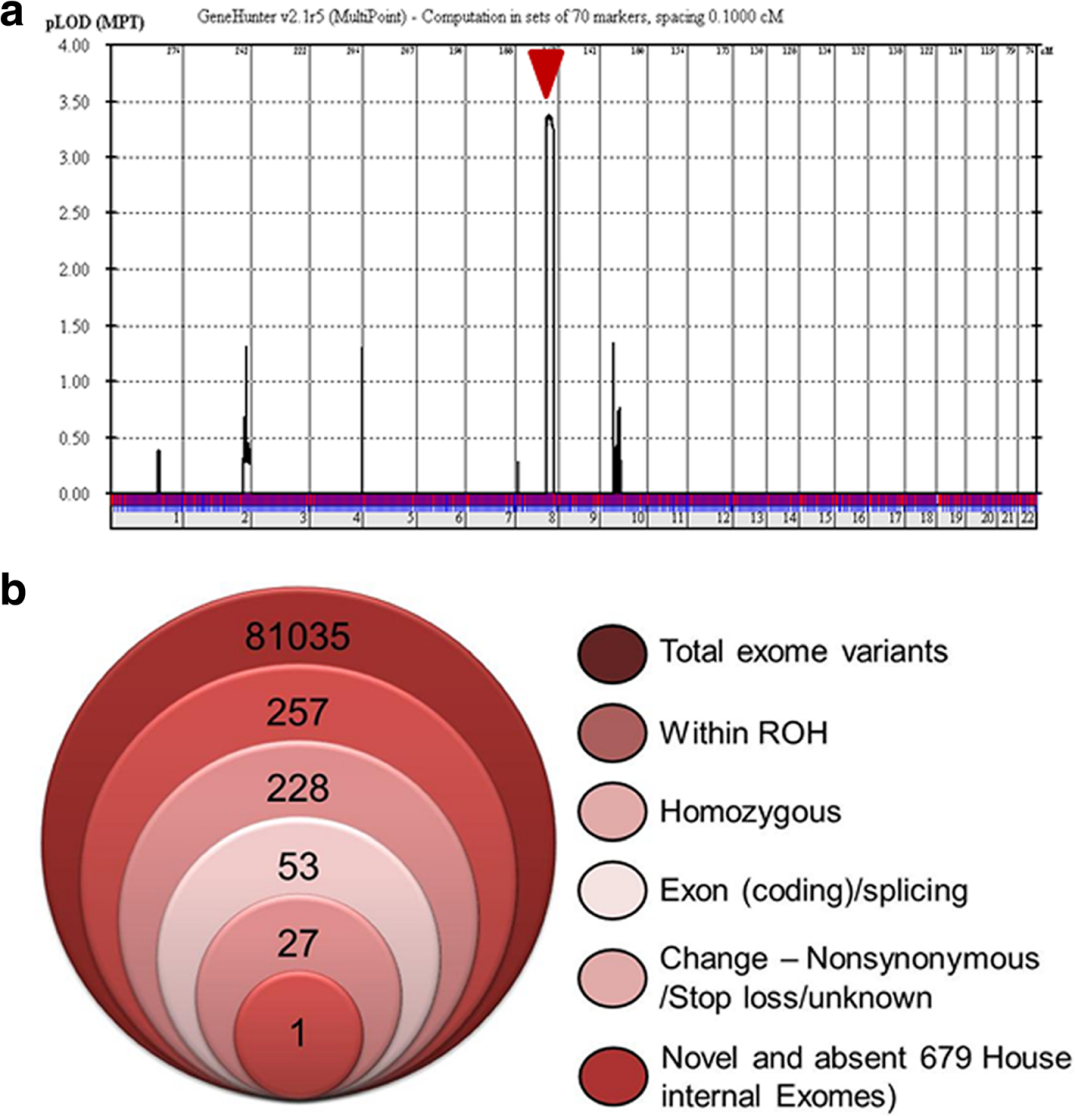
Identification of a novel genetic mutation in dilated cardiomyopathy. **a** Linkage analysis reveals a single peak indicated by the red arrow. **b** Stacked Venn diagram showing the filtering strategy to narrow down the novel locus

Given the clinical symptoms and consanguinity in the family, an autosomal recessive mode of inheritance was proposed. After signing an informed consent, blood was collected from the affected siblings, unaffected siblings, and unaffected parents, and genotyping was performed from extracted DNA. A single region of homozygosity on chromosome 8 was shared between the affected siblings (Figure S2 in Additional file 3). Linkage analysis revealed a single peak on chromosome 8 (8q24.13) with a maximum logarithm of odds (LOD) score of 3.4 (Fig. 1a) corresponding to the same ROH (Run of homozygosity) highlighted by autozygome analysis. The shared region did not include any known DCM genes. Accordingly, we performed whole exome sequencing to identify the novel locus.

### Exome sequencing identifies the pathogenic *FBXO32* variant

**Fig. 2:**
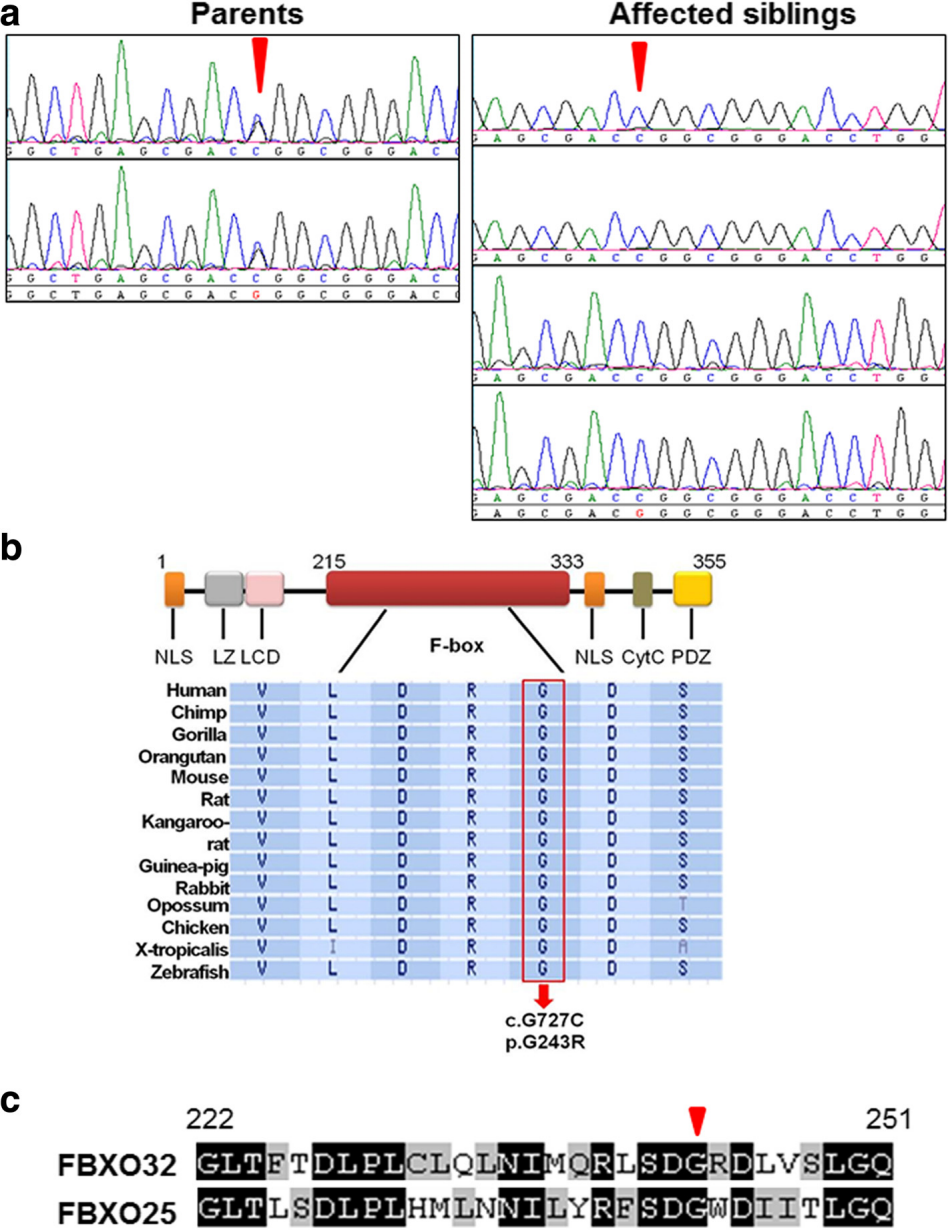
Location and conservation of the mutated amino acid. **a** DNA sequencing chromatograms showing the novel missense mutation in all affected members with the site of mutation marked by an inverted red triangle. **b** Top: Schematic representation of FBXO32 with the different domains of the protein and the G243R mutation in the F-Box domain. Bottom: multisequence alignment orthologs showing conservation of the mutation (p. G243) across species. **c** Conservation across the sub-family of human F-Box proteins. The affected amino acid residue is boxed in red. NLS = nuclear localization signals. PD = PDZ domain. LZ = leucine-zipper domain. LCD = leucine-charged residue-rich domain. F-Box = F-Box domain

Exome capture and sequencing was performed on the index patient IV.5. A summary of the raw data characteristics for the 100× coverage exome is described in Table S2 (Additional file 4). The exome data were filtered following the algorithm delineated in Fig. 1b. Only novel variants located within the ROH, homozygous, coding/splicing were considered. Applying these criteria resulted in the identification of a single novel missense variant in *FBXO32* gene NM_058229.3:c. 727G > C, p.Gly243Arg (Fig. 1c). The variant was absent from 200 ethnically matched controls as well as 679 in-house Saudi exomes and fully co-segregated with the DCM phenotype within the family as confirmed by direct Sanger sequencing (Fig. 2a). It is present at an extremely low frequency at ExAC (http://exac.broadinstitute.org/variant/14-92460188-C-T) of two heterozygotes from among 120,748 alleles for a MAF of 0.00001656. The affected amino acid residue Gly 243 was conserved across multiple species down to zebrafish and across the known F-Box protein sub-family [18] (Fig. 2b and c). The variant was predicted as pathogenic by PolyPhen-2 (probably damaging;1), MutationTaster (disease causing; 0.99), SIFT (deleterious; 0.01) and PHRED (score of 26.4). Based on these predictions and because FBXO32 has been associated with cardiac diseases in loss and gain of function studies in mice, we considered the *FBXO32* variant further for functional analyses.

### *In silico* modeling of the FBXO32 mutation predicts alteration of the SCF complex

**Fig. 3:**
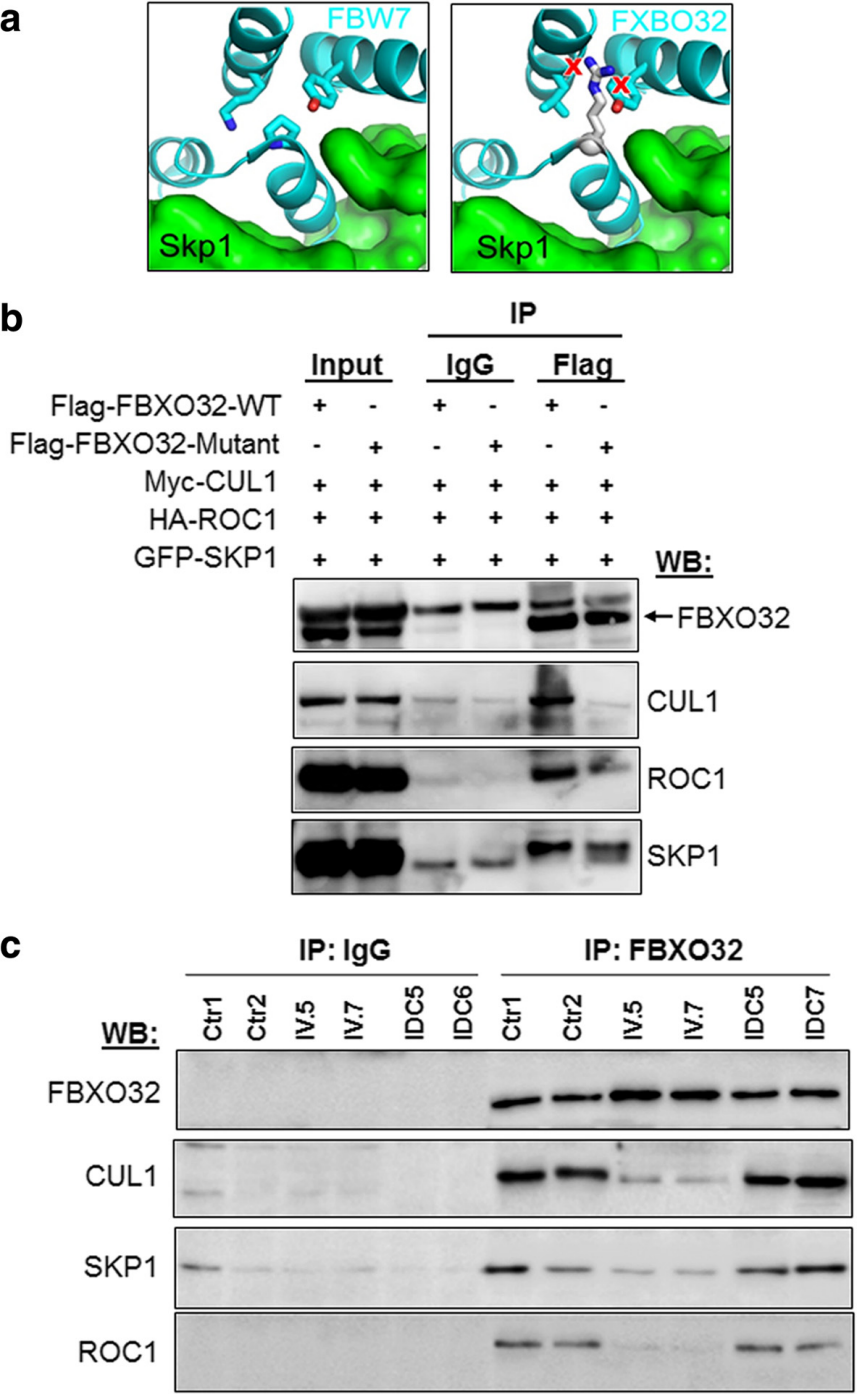
FBXO32 mutation affects the molecular interaction with components of the SCF complex. **a** Homology model of FBXO32-SKP1 interaction (right panel) based on the FBW7-SKP1 structure (left panel, PDB ID: 2OVR). FBXO32 is in ribbon representation (cyan) and SKP1 is in surface representation (green). The Gly to Arg mutation, shown as white sticks leads to clashes (marked by red crosses) with neighboring helices in the F-Box domain. **b** HEK293 cells co-transfected with the indicated plasmids. Equal amounts of protein lysates were co-immunoprecipitated with Flag resines and analyzed by immunoblotting with the indicated antibodies. Blots are representative of three independent experiments. **c** Immunoblot analysis after IP in patients IV.5 and IV.7 with FDC compared to Ctr (control) and IDC. IP was performed with control IgG antibody used a negative control

FBXO32 is part of the SCF (SKP-CUL1-F-Box) complex of ubiquitin ligases, which targets specific substrates for degradation. FBXO32 contains an F-Box domain required for proper interaction with SKP1, CUL1, and ROC1 [14, 16, 19]. Because the identified variant is located within the F-Box domain, we predicted that the interaction of FBXO32 with SKP1 might be disrupted, which may affect formation of the SCF complex and impair its function as an E3-ligase. F-Box domains are highly conserved in proteins [20]. Thus, we performed *in silico* modeling to predict the effect of the variant on the protein structure. Modeling of the F-Box domain of FBXO32 was based on the canonical structure of FBW7 (PDB ID: 2OVR) [21] using I-TASSER [22]. The FBXO32 Gly to Arg mutation (G243R) is predicted to lie on a helix at the interface between FBXO32 and SKP1 (Fig. 3a). Like the Pro residue in FBW7, the small G243 residue in FBXO32 packs tightly onto neighboring helices to maintain the correct architecture of the F-Box domain. The G to R substitution, a much larger residue, is expected to distort the fold of the F-Box as the R clashes with the surrounding helices (Fig. 3a). The outcome of the p.G243R substitution is to disrupt the proper orientation of F-Box residues interacting with SKP1, leading to a loss of interaction required for forming the E3 ubiquitin ligase, thus abrogating the function of FBXO32.

### The *FBXO32* variant impairs the formation and function of the SCF complex

CUL1 serves as a scaffolding protein that interacts with SKP1 and ROC1, while FBXO32 binds SKP1 (Figure S3 in Additional file 5) [23]. To validate the prediction of the structural modeling of the p.G243R substitution, we next assessed interaction of wild-type (WT) FBXO32 or mutant FBXO32-p.G243R with proteins of the SCF complex. WT FBXO32 or mutant FBXO32 carrying a Flag epitope were co-transfected with GFP-SKP1, myc-CUL1, and HA-ROC1 in HEK293 cells, and FBXO32 was immunoprecipitated using Flag resins followed by immunoblotting. CUL1 interacted efficiently with WT FBXO32 but not with mutant FBXO32 (Fig. 3b). ROC1 and SKP1 also interacted with WT FBXO32 whereas their binding with mutant FBXO32 was reduced (Fig. 3b). Immunoprecipitation carried out first using CUL1 absorbed on c-Myc-beads confirmed that WT FBXO32 was part of the SCF complex, but not mutant FBXO32 which failed to efficiently precipitate with CUL1. Consistent with proteins assembled in the SCF complex, CUL1/SKP1 interaction was not affected by the FBXO32 mutation (Figure S3a in Additional file 5b and c).

The F-Box domain of FBXO32 is important for the ubiquitination of specific substrates. Thus, we evaluated the effect of the FBXO32 mutation on global ubiquitination of cellular proteins. Cells expressing mutant FBXO32 displayed reduced ubiquitination of cellular proteins compared to cells expressing WT FBXO32 (Figure S4a in Additional file 6). Furthermore, expression of mutant FBXO32 in cardiomyocytes stabilized two known substrates of FBXO32, CHMP2B, and Calcineurin A (Figure S4b in Additional file 6).

Next we assessed FBXO32/SKP1/CUL1/ROC1 interaction in the heart of patients IV.5 and IV.7 with FDC collected at the time of cardiac transplantation using immunoprecipitation. We also evaluated interaction in control hearts and in the hearts of patients with idiopathic dilated cardiomyopathy (IDC). Strong binding of FBXO32 with CUL1 was detected in control hearts. Strikingly, FBXO32/CUL1 interaction was reduced in hearts IV.5 and IV.7 but not in control hearts and in the hearts of patients with IDC (Fig. 3c). Binding of FBXO32 with ROC1 and SKP1 was also reduced in hearts IV.5 and IV.7 but not in the heart of patients with IDC (Fig. 3c). Together, these results indicate that the *FBXO32* mutation affects interaction of FBXO32 with CUL1, ROC1, and SKP1, thus impairing proper formation of the SCF complex.

### The autophagy/lysosome system is impaired in the hearts of the patients with the *FBXO32* mutation

FBXO32 regulates the stability of calcineurin A [13], an important mediator of cardiac hypertrophy [24] (for Review). IkB-α is also targeted for degradation by FBXO32 [15]. Thus we evaluated calcineurin A and IkB-α protein expression in the control hearts, in hearts IV.5 and IV.7 from our recruited family, in a dilated heart of an unrelated family, and in IDC. Calcineurin A was upregulated in hearts IV.5 and IV.7 and in the majority of the diseased hearts (Figure S5 in Additional file 7), showing that altered expression of calcineurin A is a common response in dilated cardiomyopathy [25]. IkB-α protein level was similar in the hearts IV.5 and IV.7 and in the control hearts, suggesting that the *FBXO32* mutation does not regulate IkB-α expression.

**Fig. 4:**
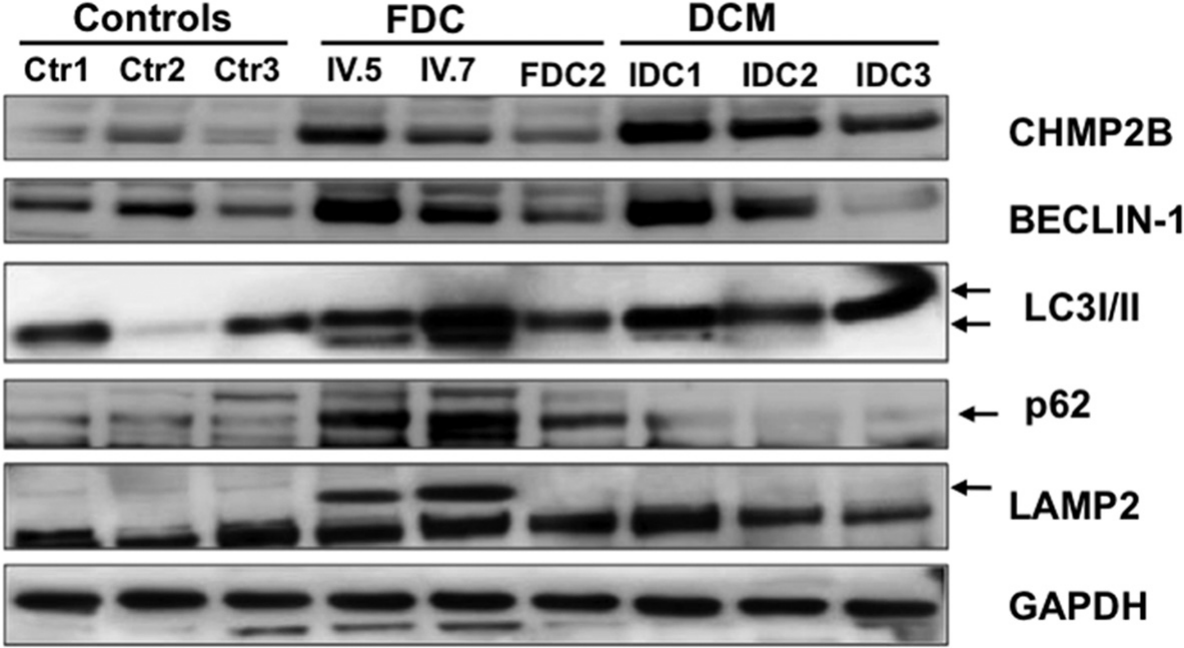
The *FBXO32* mutation impairs the expression of selected proteins of the autophagy/lysosomal system. Immunoblot blot analysis of the indicated autophagy and lysosomal marker in FDC patients IV.5 and IV.7 carrying the *FBXO32* variant, in a cardiomyopathic heart from another family (FDC2), in idiopathic dilated hearts (IDC) and in control hearts (Ctr)

FBXO32 has recently been shown to regulate the turnover of the charged multivesicular body protein 2B (CHMP2B), an essential mediator for autophagosome-lysosome fusion [17]. Based on this, we assessed CHMP2B protein expression in our collection of hearts, as well as the expression of the common autophagy marker BECLIN-1. Immunoblot analysis showed that CHMP2B and BECLIN-1 protein expression were induced to variable degree in patient hearts IV.5 and IV.7 and in the other diseased hearts, indicating a similar response in cardiomyopathy of familial and idiopathic origin (Fig. 4). Next, we assessed levels of the microtubule-associated protein1 light chain 3 (LC3) and the conversion of LC3-I to LC3-II which is an indicator of autophagic activity [26]. Increased LC3-II was detected in hearts IV.5 and IV.7 while it was almost undetectable in the other familial heart and in the hearts of patients with IDC, suggesting a block of autophagic flux in the heart of patients carrying the *FBXO32* mutation. Consistent with this, expression of p62 (sequestosome 1, SQSTM1), a selective autophagy substrate that accumulates when autophagy is inhibited, increased more strongly in the hearts of patients IV.5 and IV.7 compared to the other diseased hearts [27]. Strikingly, expression level of the lysosome-associated membrane protein-2 (LAMP2), a marker of end-stage autophagy, strongly increased only in hearts IV.5 and IV.7 but not in the other cardiomyopathic hearts. All together, these results suggest that although autophagy is induced in the hearts of the patients with the *FBXO32* mutation, the execution of autophagy is not complete.

## Discussion

DCM is a disease of the myocardium causing left ventricular enlargement and systolic dysfunction progressing to heart failure. Rare variants in more than 30 genes have been reported to cause FDC most of which encoding proteins of the sarcomere. However, the majority of the DCM-causing mutations still remain to be identified and the prevalence of DCM is believed to be largely underestimated [4]. New variants emerging from a large population-based study using exome data from the NHLBI/GO Exome Sequencing Project indicate an over-representation of cardiomyopathy-associated variants [28]. In this study, we identify *FBXO32* as a new locus causing DCM. Evidence that the *FBXO32* mutation is disease-causing is supported by the positional mapping showing that the four affected patients in this large family carry the homozygous mutation. Furthermore, the modeling and functional data show that the mutation causes a defect in the SCF complex, which is recapitulated in the two hearts of the patients with FDC. Finally, our molecular analysis showed impaired expression of “late” autophagy markers in the two FDC hearts but not in other cardiomyopathic hearts of familial and idiopathic origin. These changes were also observed in a mouse model of FBXO32 deficiency. Although we cannot exclude the possibility that a non-genic mutation may contribute to the pathogenesis of DCM in this family, our exome sequencing data combined with the modeling of the mutation and our functional analysis provide compelling evidence that the *FBXO32* variant is pathogenic.

DCM was initially suspected in the family from the history of cardiac transplantation in three of the affected patients. The fourth sibling was also recommended for heart transplantation. FDC can be asymptomatic for years or have incomplete penetrance, which can represent a challenge for accurate clinical diagnosis. Also, variable penetrance and expression of the disease can complicate DCM assessment. In our study, full clinical evaluation including echocardiography was done by a trained cardiologist who confirmed an early onset of the disease in the four affected siblings.

Homozygosity for the *FBXO32* mutation segregated with the affected status of the siblings in the family. Because most of the DCM-causing mutations are rare, we screened 679 in-house Saudi exomes and performed Sanger sequencing from 200 ethnically-matched controls. The variant was predicted to have a damaging effect on FBXO32 protein structure according to four different web-based programs and was found at an extremely low frequency (MAF of 0.00001656). These features together with the high conservation of the affected amino acid across species and within the sub-family of F-BOX proteins are consistent with the variant being disease-causing.

FBXO32 is a component of the SCF family of ubiquitin ligases. The F-Box domain of FBXO32 functions as an adaptor that binds SKP1 and thereby CUL1 and ROC1 [29]. Because the G243R variant is located in the F-Box domain, we hypothesized that the mutation may impair proper assembly of the SCF and affect its function. Modeling of the variant from the crystal structure of FBW7 predicted loss of interaction between FBXO32 and SKP1. Our co-precipitation experiments showed that an intact F-Box is critical for FBXO32 to bind other members of the SCF as interaction with CUL1 was strongly impaired when G243 was mutated to R. Furthermore, expression of mutant FBXO32 in cells resulted in a general ubiquitination defect. Most importantly, interaction of FBXO32 with SKP1 was disrupted in the two hearts of the patients carrying the *FBXO32* mutation while interaction was intact in control and IDC hearts. These results indicate that the mutation in the F-Box domain of *FBXO32* disrupts binding of several key proteins of the SCF complex causing dysregulated degradation of specific cellular proteins.

Gain and loss of function studies in transgenic and knockout mice revealed a role of FBXO32 in maladaptive cardiac remodeling through regulation of calcineurin A and IkB-α [15, 16]. Thus, we thought to evaluate calcineurin A and IkB-α expression in the heart of the patients with the *FBXO32* mutation. In agreement with previous reports [30], calcineurin A was induced in human heart failure. However, calcineurin A was increased in all cardiomyopathic hearts of familial or idiopathic etiology, suggesting that stabilization of calcineurin A is a common feature of end-stage heart failure rather than a unique characteristic of the *FBXO32* mutation. IkB-α protein expression was similar in control hearts and in the hearts of the two patients carrying the *FBXO32* mutation, also ruling out that stabilization of IkB-α is a major contributor to the cardiomyopathy in the affected patients.

The recent finding that FBXO32 deficiency causes an aging-related cardiomyopathy in mice due to impaired autophagy provided the first direct link between FBXO32 and the autophagy system [17]. This study prompted us to evaluate the role of this conserved catabolic pathway in the pathogenesis of the FDC. Our biochemical analysis showed increased expression of selected proteins implicated in the “late” stage of the autophagy system in the two hearts with the *FBXO32* mutation. Indeed, we detected a remarkable accumulation of LAMP2 in the hearts of the patients carrying the *FBXO32* variant but not in the heart of another patient with FDC or in other DCM hearts. Since there is ample documentation between dysregulation of the autophagic flux and the development of heart failure and of other cardiovascular diseases [31–33], our data suggest a late-stage inhibition in autophagosome processing in the affected patients. Thus, the defect in the last step of the autophagic pathway observed in the human hearts with the *FBXO32* mutation, which is also recapitulated in aging FBXO32-deficient mice, is likely to contribute to the cardiomyopathy.

## Conclusion

**Fig. 5:**
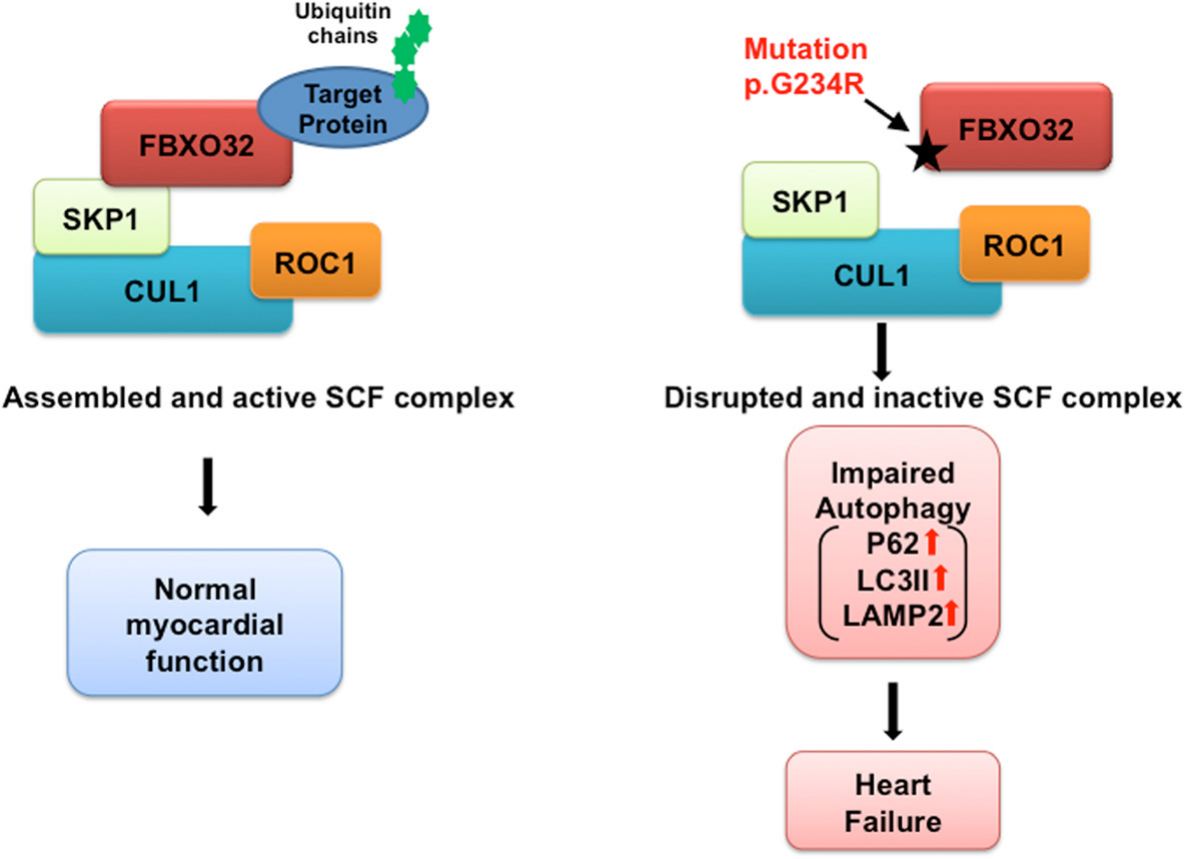
Hypothetical model of impaired autophagy flux mediated by the mutated FBXO32. **a** SCF complex containing FBXO32 induces the ubiquitination and subsequent degradation of target proteins. **b** Mutation in FBXO32 results in an inactive SCF complex, which stabilizes selective proteins regulating autophagy resulting in heart failure

Overall, our study identifies *FBXO32* as a new locus for recessive DCM. Our biochemical analysis revealed that the mutation alters the formation and function of the SCF complex. Assessment of FBXO32 molecular targets in cardiac tissue of the affected individual suggests that the *FBXO32* mutation causes cardiomyopathy through impaired expression of selective proteins of the autophagy system (Fig. 5). This study may open new diagnostic testing for patients with FDC with potential new windows for disease treatment.

## Methods

### Patient recruitment

The family is from Saudi Arabia. Patients were evaluated by a board-certified cardiologist. All participants provided written informed consent under protocols approved by the Institutional Review Board at King Faisal Specialist Hospital & Research Centre (RAC#2100 024 and #2010 020). Further details can be provided to researchers upon request. The study was carried out in accordance with the principles of the Declaration of Helsinki.

### Human hearts

Control donor hearts were from patients with no sign of cardiac disease whose hearts could not be transplanted due to ABO mismatch. The hearts of one patient from another family with DCM was collected as an additional control. Three patients with idiopathic dilated cardiomyopathy (IDC) were collected at the time of cardiac transplantation. We were also able to collect the hearts of two of the affected siblings in our family, patients IV.5 and IV.7. Written informed consent was obtained for all the patients that underwent heart transplantation after IRB approval (RAC# 2100 024). The hearts were carefully dissected in the same anatomical regions, and tissues from the left ventricle were used for biochemical and histological analyses.

### Echocardiography

Echocardiography was performed for patients IV.5 and IV.7 prior to the cardiac transplantation. The other affected siblings were also evaluated by echocardiography to assess the presence or absence of DCM.

### DNA extraction, genotyping, linkage analysis, and homozygosity mapping

Genomic DNA was extracted from venous blood collected from all the family members. Genome-wide genotyping was performed using the Axiom SNP ChIP platform (Affymetrix, Santa Clara, CA, USA) followed by AutoSNPa software used to identify homozygous intervals of more than 1 Mb in length (http://dna.leeds.ac.uk/autosnpa/). Linkage analysis was performed using Easy Linkage software and multipoint LOD scores were calculated assuming an autosomal recessive mode of transmission.

### Exome sequencing

Whole-exome sequencing was performed using TruSeq Exome Enrichment kit (Illumina, San Diego, CA, USA). Exome capture was performed according to the Illumina protocols using TruSeq Exome Enrichment kit (Illumina). Briefly, samples were prepared as an Illumina sequencing library, and in the second step, the sequencing libraries were enriched for the desired target using the Illumina Exome Enrichment protocol. The captured libraries were sequenced using the Illumina HiSeq 2000 Sequencer. The reads were mapped by BWA (http://bio-bwa.sourceforge.net/) against UCSC hg19 (http://genome.ucsc.edu/). The SNPs and Indels were detected by SAMTOOLS (http://samtools.sourceforge.net/). The variant Databases dbSNP and 1000G were used to determine the variant frequency. The full detail of the data coverage and sequencing depth can be found in Additional file 4 Table S2.

Variants from WES were filtered such that only novel, coding/splicing, homozygous variants that are within the autozygome shared between the affected in the family and not present in the 679 in-house Saudi exomes as described before [34]. Variants remaining after this filtering process were prioritized based on being predicted as disease causing by pathogenicity predictor tools (Mutation taster (http://www.mutationtaster.org/); Polyphen2 (http://genetics.bwh.harvard.edu/pph2/)), SIFT (http://sift.jcvi.org/), and CADD score (http://cadd.gs.washington.edu/).

### PCR

Variants of interest were validated by PCR amplification of the variant-containing exon from the original patient sample, followed by Sanger sequencing. Genes located within the ROH (Runs of homozygosity) which were not fully covered by the exome sequencing, were also directly sequenced using Sanger sequencing.

### Site-directed mutagenesis of FBXO32

The FBXO32 mutation was generated by site-directed mutagenesis using QuikChange II Site-Directed Mutagenesis Kit (Agilent Technologies). Overlapping PCR was used to introduce a site-specific point mutation. The used primers were: 5′- GCAGAGGCTGAGCGAC(C)GGCGGGACCTGGTCAG -3′ and 5′- CTGACCAGGTCCCGCCGGTCGCTCAGCCTCTGC -3′. The base change (G to C) in the relevant nucleotide is shown in parentheses. Each PCR reaction (50 μL) contained 50 ng FBXO32-MYC-DDK plasimd, 5 μL 10× reaction buffer, 1.5 μL dNTPs, 125 ng forward primer and 125 ng reverse primer, and 2.5 U Pfu ultra High-Fidelity DNA polymerase. The thermal cycle program was 95 °C/5 min, followed by 18 cycles of (95 °C/50 s, 55 °C/50 s, 68 °C/7 min) and a final extension step of 68 °C for 7 min. Afterwards the PCR product was treated with DpnI for 1 h at 37 °C and transformed into XL1-Blue competent cells provided by the kit. The mutagensis product was verified by sequencing.

### Cell culture

HEK293 cells were maintained in DMEM (10%FCS, 1 % Pen/Strep). Primary neonatal rat cardiomyocytes were isolated using the Worthington Neonatal Cardiomyocyte Isolation System (Worthington Biochemical Corp, USA) following the manufacturer’s instruction.

### Transfection of cells

Twenty-four hours before transfection, cells were seeded to be 60–70 % confluent at the time of transfection. The next day the transfection was performed using Lipofectamine® 2000 (Invitrogen™, USA) according to the manufacturer’s protocol. HEK 293 Cells were co-transfected with WT FBXO32 or mutant FBXO32 carrying a Flag epitope, Myc-tagged CUL1 and GFP-SKP1. Forty-eight hours post transfection, total cell lysates were prepared and analyzed by immunoblotting or subjected to immunoprecipitation. Isolated primary neonatal rat cardiomyocytes were transfected 12 h after isolation with the indicated plasmids using Lipofectamine® 2000 (Invitrogen™, USA) according to the manufacturer’s protocol. 16 hours post transfection, media was changed. Forty-eight hours post transfection, cell lysates were prepared and analyzed by immunoblotting using the indicated antibodies.

### Plasmids and antibodies

Eukaryotic expression vectors for FBXO32 carrying a Flag epitope (Flag-FBXO32), Myc-CUL1, and GFP-SKP1 were purchased from Origene. Antibodies against FBXO32, cMyc, CUL1, SKP1, HA (Hemagglutinin), GAPDH, and ROC-1 were purchased from Santa Cruz. BECLIN-1, CHMP2B, LAMP2, FBXO32 (mAB), LC3a/B, and p62 were purchased from Abcam, calcineurin A and IkB-α were from Cell Signaling, and IkB-α was from Abcam.

### Protein extraction and immunoblot analysis

Protein extracts from human heart tissue were prepared by grinding the tissue in liquid nitrogen followed by homogenization using sonication in a lysis buffer supplemented with protease inhibitor cocktail. After centrifugation at 12,000 rpm at 4 °C for 10 min, supernatants were recovered and used for immunoblot or immunoprecipitation analyses. Thirty micrograms of protein from tissue or whole cell extracts were separated on 4–12 % NuPAGE® Novex® Bis-Tris Gels (Invitrogen, USA) and transferred onto nitrocellulose membrane. After blocking of the membranes, the indicated primary antibodies were incubated overnight at 4 °C. Secondary reactions were performed for 1 h and after washing, chemiluminescence was performed and the signals were visualized using LAS 4000 analyzer (GE) and analyzed by ImageQuant software. For immunoprecipitation, 400–700 μg of total cell lysates were incubated with Flag- or Myc-resins (Sigma) coupled with primary antibody or control IgG in a buffer supplemented with protease inhibitors for 2 h at 4 °C. After centrifugation, the beads were washed and after elution, proteins were resolved by SDS-PAGE and immunoblotting was performed as described before.

## Availability of supporting data

The exome file is kept a searchable and secured access database that can be accessed at http://shgp.kfshrc.edu.sa/bioinf/db/variants/dg. The username and password can be given upon request.

### Funding

This work was supported in part by a grant from King Abdulaziz City for Science and Technology (KACST # 10-BIO1350-20) awarded to CP and by institutional funding from King Faisal Specialist Hospital & Research Centre.

### Ethics

The study was carried out in accordance with the principles of the Declaration of Helsinki.

## Additional files

Additional file 1: Table S1. Clinical characteristics of the studied family. (DOCX 82 kb) Additional file 2: Figure S1. Echocardiographic images of patients from the studied family. Echocardiography performed on two of the affected siblings index patients IV.5 (a) and IV.7 (b) prior to heart transplantation. M-mode echo and parasternal long-axis view showing dilatation of the left ventricle and of the left atria with severe hypokinesia. (TIF 5479 kb) Additional file 3: Figure S2. Autozygome analysis in the studied family. Autozygosity mapping was performed using AgileMultideogram. The result is showing the single shared ROH (runs of homozygosity) on chromosome 8 between the four affected members (dark blue). (TIF 4051 kb) Additional file 4: Table S2. Coverage and sequencing depth of the exome data. (DOCX 17 kb) Additional file 5: Figure S3. Mutation in FBXO32 abrogates binding to SKP1 within the SCF complex. **a** Schematic representation of the SCF complex. The FBXO32 mutation represented as a black triangle is within the F-Box domain which normally mediates binding to SKP1. **b** Immunoprecipitation showing reduced interaction of mutant FBXO32 with CUL1 after co-transfection of HEK293 cells with WT-FBXO32 or mutant-FBXO32 and CUL1, ROC1, and SKP1. **c** Quantitative analysis of (b) from three independent experiments. (TIF 2185 kb) Additional file 6: Figure S4. Ubiquitination defect in cells expressing mutant FBXO32. **a** Co-immunopricipitation analysis. HEK293 cells were transfected with the indicated plasmids and immunoblot analysis was performed from total cell lysates using a specific anti-ubiquitin antibody. FBXO32 expression is shown as well as GAPDH. The blot is representative of three independent experiments. **b** Immunoblot analysis of the ubiquitination in cardiomyocytes. Cells were transfected with the Flag-FBXO32-WT or Flag-FBXO32-Mutant and whole cell extracts were analyzed by immunoblotting using the indicated antibodies. (TIF 1928 kb) Additional file 7: Figure S5. Expression of the FBXO32 substrates calcineurin A and IkB-α in FDC and IDC. Immunoblot analysis showing calcineurin A and IkB-α protein expression in control hearts, in the heart of patients IV.5 and IV.7 carrying the *FBXO32* variant, in a cardiomyopathic heart from another family (FDC2) and in idiopathic dilated hearts (IDC). FBXO32 and GAPDH are also shown. (TIF 3699 kb)

## Abbreviations

Ctr: Control
DCM: Dilated cardiomyopathy
FDC: Familial dilated cardiomyopathy
IDC: Idiopathic dilated cardiomyopathy
LA: Left atria
LOD: Maximum logarithm of odds
LV: Left ventricle
RA: Right atria
ROH: Runs of homozygosity
RV: Right ventricle
