# Supplementary material for: FBXO32, encoding a member of the SCF complex, is mutated in dilated cardiomyopathy
Source: Genome Biol. 2016 Jan 11;17:2. doi: 10.1186/s13059-015-0861-4 (PMC4707779; doi:10.1186/s13059-015-0861-4)
Supplement: Additional file 1: Table S1. — Clinical characteristics of the studied family. (DOCX 82 kb) [file 13059_2015_861_MOESM1_ESM.docx]

**Additional file1:**

**Table S1: Clinical characteristics of the family**

| **Subject** | **DCM** | **LVIDd (cm)** | **RVDd (cm)** | **IVSd (cm)** | **LVPWd (cm)** | **EF (%)** | **Mutation present** | **Comments** |
| --- | --- | --- | --- | --- | --- | --- | --- | --- |
| III.1 | No |  |  |  |  |  | Heterozygous | No symptoms |
| III.2 | No |  |  |  |  |  | Heterozygous | No symptoms |
| IV.1 | No |  |  |  |  |  | Heterozygous | No symptoms |
| IV.2 | No |  |  |  |  |  | Heterozygous | No symptoms |
| IV.3 | No |  |  |  |  |  | Heterozygous | No symptoms |
| IV.4 | Yes | 6.3 | - | 0.86 | 0.75 | <25 | Homozygous | Died of advanced heart failure, refused heart transplantation |
| IV.5 | Yes | 6.8 | 3.7 | 0.73 | 0.73 | <25 | Homozygous | Heart transplant |
| IV.6 | No |  |  |  |  |  | No | No symptoms |
| IV.7 | Yes | 7.1 | 3.3 | 0.67 | 1.3 | 15 | Homozygous | Heart transplant |
| IV.8 | Yes | 6.3 | 3.6 | 0.49 | 0.64 | 10 | Homozygous | Heart transplant, |
| IV.9 | No |  |  |  |  |  | Heterozygous | No symptoms |
| IV.10 | No |  |  |  |  |  | Heterozygous | No symptoms |

**Abbreviations**: DCM: dilated cardiomyopathy; EF: ejection fraction; IVSd: inter-ventricular septum thickness at diastole; LA: left atria; LV: left ventricle; LVIDd: left ventricular internal diameter at diastole; LVPWd: left ventricle posterior wall thickness at diastole; M: male; RV: right ventricle; RVDd: right ventricular diameter at diastole.
