# Supplementary figures and images for: FBXO32, encoding a member of the SCF complex, is mutated in dilated cardiomyopathy
Source: Genome Biol. 2016 Jan 11;17:2. doi: 10.1186/s13059-015-0861-4 (PMC4707779; doi:10.1186/s13059-015-0861-4)

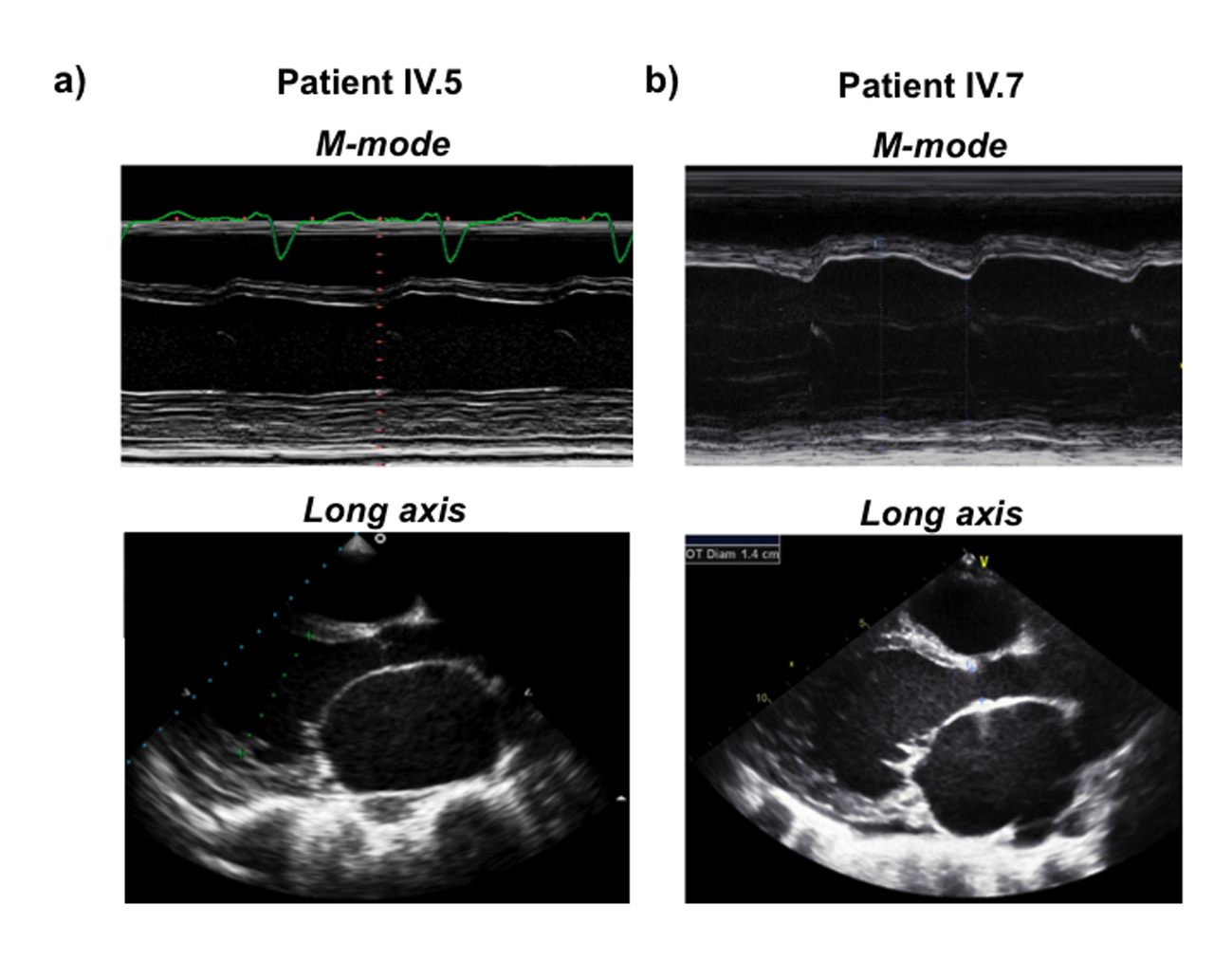

Supplement: Additional file 2: Figure S1. — Echocardiographic images of patients from the studied family. Echocardiography performed on two of the affected siblings index patients IV.5 (a) and IV.7 (b) prior to heart transplantation. M-mode echo and parasternal long-axis view showing dilatation of the left ventricle and of the left atria with severe hypokinesia. (TIF 5479 kb) [file 13059_2015_861_MOESM2_ESM.tif]

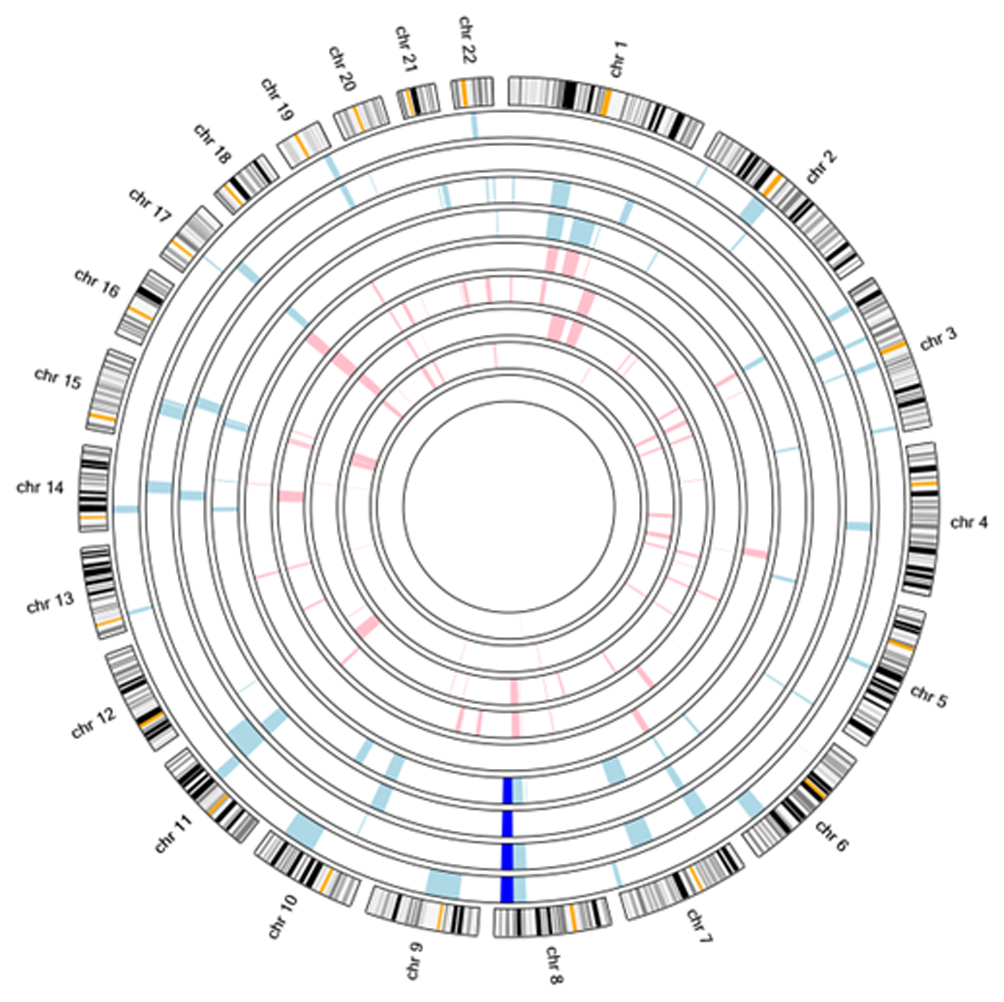

Supplement: Additional file 3: Figure S2. — Autozygome analysis in the studied family. Autozygosity mapping was performed using AgileMultideogram. The result is showing the single shared ROH (runs of homozygosity) on chromosome 8 between the four affected members (dark blue). (TIF 4051 kb) [file 13059_2015_861_MOESM3_ESM.tif]

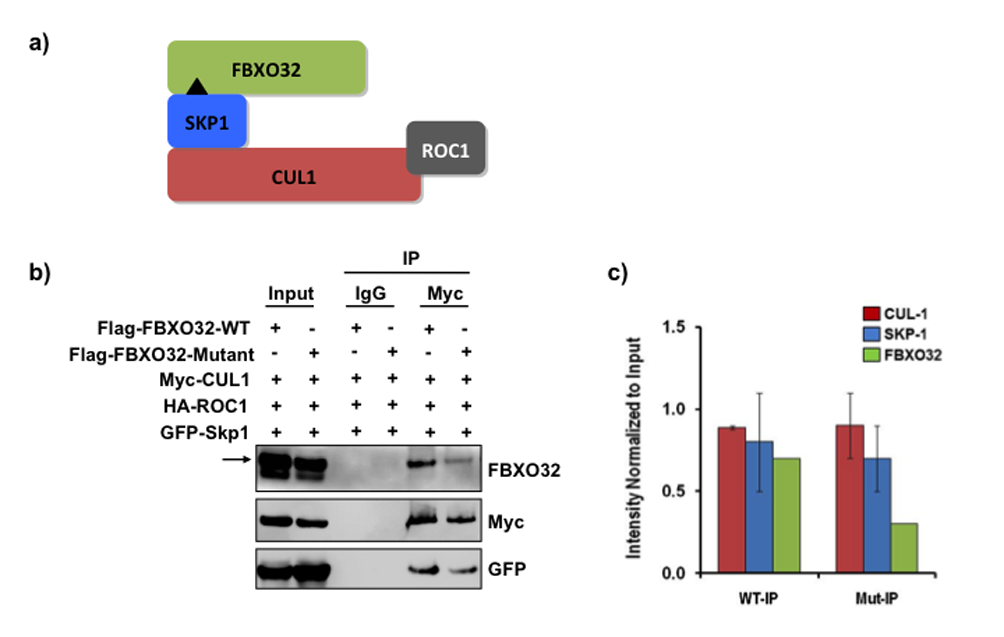

Supplement: Additional file 5: Figure S3. — Mutation in FBXO32 abrogates binding to SKP1 within the SCF complex. a Schematic representation of the SCF complex. The FBXO32 mutation represented as a black triangle is within the F-Box domain which normally mediates binding to SKP1. b Immunoprecipitation showing reduced interaction of mutant FBXO32 with CUL1 after co-transfection of HEK293 cells with WT-FBXO32 or mutant-FBXO32 and CUL1, ROC1, and SKP1. c Quantitative analysis of (b) from three independent experiments. (TIF 2185 kb) [file 13059_2015_861_MOESM5_ESM.tif]

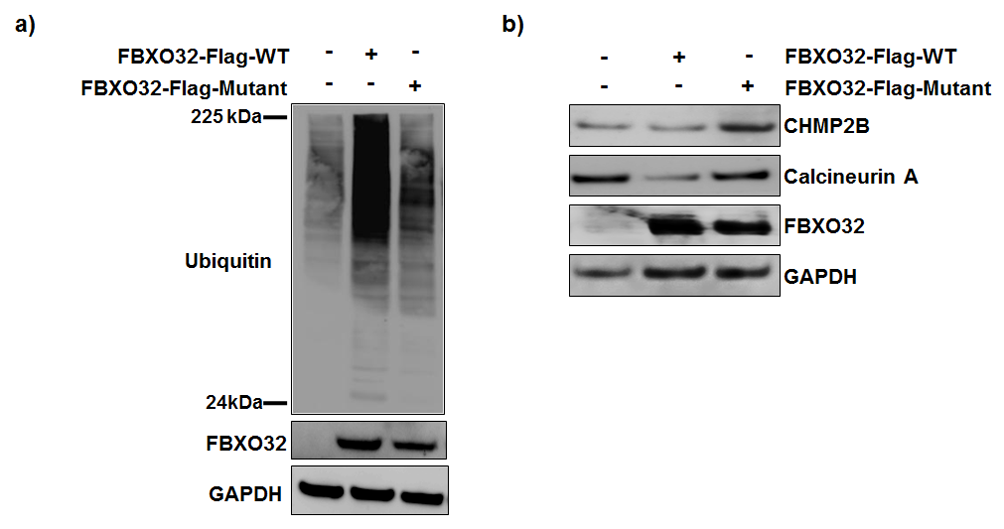

Supplement: Additional file 6: Figure S4. — Ubiquitination defect in cells expressing mutant FBXO32. a Co-immunopricipitation analysis. HEK293 cells were transfected with the indicated plasmids and immunoblot analysis was performed from total cell lysates using a specific anti-ubiquitin antibody. FBXO32 expression is shown as well as GAPDH. The blot is representative of three independent experiments. b Immunoblot analysis of the ubiquitination in cardiomyocytes. Cells were transfected with the Flag-FBXO32-WT or Flag-FBXO32-Mutant and whole cell extracts were analyzed by immunoblotting using the indicated antibodies. (TIF 1928 kb) [file 13059_2015_861_MOESM6_ESM.tif]

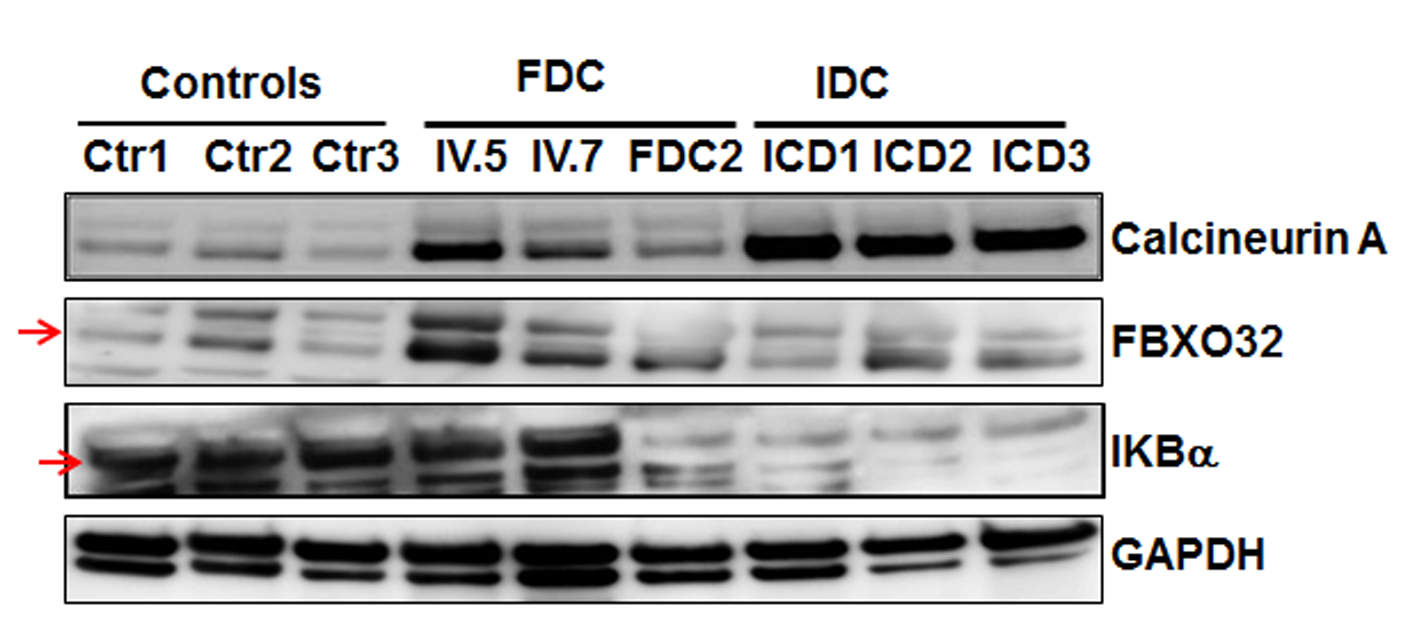

Supplement: Additional file 7: Figure S5. — Expression of the FBXO32 substrates calcineurin A and IkB-α in FDC and IDC. Immunoblot analysis showing calcineurin A and IkB-α protein expression in control hearts, in the heart of patients IV.5 and IV.7 carrying the FBXO32 variant, in a cardiomyopathic heart from another family (FDC2) and in idiopathic dilated hearts (IDC). FBXO32 and GAPDH are also shown. (TIF 3699 kb) [file 13059_2015_861_MOESM7_ESM.tif]
