# Supplementary material for: FBXO32, encoding a member of the SCF complex, is mutated in dilated cardiomyopathy
Source: Genome Biol. 2016 Jan 11;17:2. doi: 10.1186/s13059-015-0861-4 (PMC4707779; doi:10.1186/s13059-015-0861-4)
Supplement: Additional file 4: Table S2. — Coverage and sequencing depth of the exome data. (DOCX 17 kb) [file 13059_2015_861_MOESM4_ESM.docx]

**Additional file 4**

**Table S2. Summary of the raw data from whole-exome Sequencing**

**(100x coverage).**

| **Sample Name** | **CRP1-1582** |
| --- | --- |
| **Total reads** | 50,086,032 |
| **Total yield (bp)** | 5,058,689,232 |
| **Read length (bp)** | 101,0 |
| **Target regions (bp)** | 62,085,286 |
| **Average throughput depth of target regions** | 81,5 |
| **Initial mappable reads (mapped to human genome)** | 49,986,286 |
| **% Initial mappable reads (out of total reads)** | 99.80% |
| **Non-redundant reads (de-duplicated by Picard tools)** | 44,214,656 |
| **% Non-redundant reads (out of initial mappable reads)** | 88,5% |
| **Non-redundant unique reads (uniquely mapped to human genome)** | 38,505,809 |
| **% Non-redundant unique reads (out of non-redundant reads)** | 87,1% |
| **On-target reads (mapped to target regions)** | 24,857,232 |
| **% On-target reads (out of non-redundant unique reads)** | 64,6% |
| **% Coverage of target regions (more than 1X)** | 95,0% |
| **Number of on-target genotypes (more than 1X)** | 58,970,505 |
| **% Coverage of target regions (more than 10X)** | 86,9% |
| **umber of on-target genotypes (more than 10X)** | 53,921,382 |
| **Mean read depth of target regions** | 32,4 |
| **Number of SNPs** | 73,765 |
| **Number of coding SNPs** | 20,623 |
| **Number of synonymous SNPs** | 10,623 |
| **Number of nonsynonymous SNPs** | 9,478 |
| **Number of Indels** | 7,270 |
| **Number of coding Indels** | 398 |
